# Hybrid Integrated Platforms for Silicon Photonics

**DOI:** 10.3390/ma3031782

**Published:** 2010-03-12

**Authors:** Di Liang, Gunther Roelkens, Roel Baets, John E. Bowers

**Affiliations:** 1 Department of Electrical and Computer Engineering, University of California, Santa Barbara, CA, 93106, USA; E-Mail: bowers@ece.ucsb.edu (J.E.B.); 2 Photonics Research Group, IMEC - Ghent University, Sint-Pietersnieuwstraat 41, B-9000 Ghent, Belgium; E-Mails: gunther.roelkens@intec.ugent.be (G.R.); roel.baets@intec.ugent.be (R.B.)

**Keywords:** hybrid integration, wafer bonding, silicon photonics

## Abstract

A review of recent progress in hybrid integrated platforms for silicon photonics is presented. Integration of III-V semiconductors onto silicon-on-insulator substrates based on two different bonding techniques is compared, one comprising only inorganic materials, the other technique using an organic bonding agent. Issues such as bonding process and mechanism, bonding strength, uniformity, wafer surface requirement, and stress distribution are studied in detail. The application in silicon photonics to realize high-performance active and passive photonic devices on low-cost silicon wafers is discussed. Hybrid integration is believed to be a promising technology in a variety of applications of silicon photonics.

## 1. Introduction

Prior to the invention of integrated circuits, methods were developed to integrate different materials, aiming to utilize advantages from each material simultaneously. This idea was advanced along with the progress of semiconductor industry. Wafer bonding represents one of oldest and the most important approaches to realize this goal, especially when its basic criteria: clean, mirror-polished, flat surfaces [1] become easy to meet today. Present microelectronic circuit integration on silicon substrates is reaching practical bottlenecks, primarily in data transmission bandwidth and power consumption [2,3]. Introducing optics into conventional silicon microelectronics. is believed to be the future path of integrated circuits [3] and other new emerging applications for silicon photonics. Wafer bonding has also found its new application in these technology revolutions besides demonstrated examples, such as hybrid light emitting diodes, vertical-cavity surface-emitting lasers, photodetectors, optical micro-electro-mechanical systems, and sensors, *etc.* [4]. 

Integration of GaAs and InP, the flagship substrate materials of photonics, and silicon, the undisputed material of choice in electronics, has been well studied. While they have naturally been attractive material candidates for integration over decades, they have only rarely been successfully integrated together. Physically, the large mismatch in lattice constant and thermal expansion coefficient (TEC) make monolithic integration very difficult. Wafer bonding-based hybrid integration [4] is not limited by lattice mismatch but still needs to tackle the TEC mismatch issue.

In this paper we review three *low-temperature* wafer bonding techniques that have been used to demonstrate InP-on-silicon hybrid platform and heterogeneous platform, both recently enabling high-performance photonic devices. The first two bonding methods are O_2_ plasma-assisted and SiO_2_ covalent direct bonding, both sharing similar bonding mechanism and falling into molecule (or hydrophilic) bonding category. Since only inorganic materials are involved in the integration process, we discuss them in the inorganic-to-inorganic bonding section (section 2). The third method uses polymer as an adhesive to “glue” silicon and III-V wafers together, and will be discussed in the organic-to-inorganic bonding section (section 3). Finally, two hybrid platforms for silicon photonic applications are briefly introduced in section 4. For more detail information on these platforms please see references [5,6].

## 2. O_2_ Plasma-Assisted/SiO_2_ Covalent Direct Bonding 

For conventional direct bonding, high-temperature is typically required to strengthen the bonding. It is therefore often referred to as “fusion bonding”. In other bonding applications, this has proven highly effective, however special process development is required when a high-temperature anneal is strictly prohibited in III-V-to-silicon bonding. O_2_ plasma surface treatment emerged as an attractive approach to obtain high bonding strength under a low-temperature (<400 °C) anneal [7,8]. Its bonding mechanism is discussed below. SiO_2_ covalent bonding, the dominant process to fabricate microelectronics-grade silicon-on-insulator (SOI) wafers up to 300 mm [9], is a relatively old approach, but with careful surface treatment [10,11,12,13], can also be modified to meet the same low temperature, high strength criterion. 

### 2.1. Bonding process flow and mechanism

**Figure 1:**
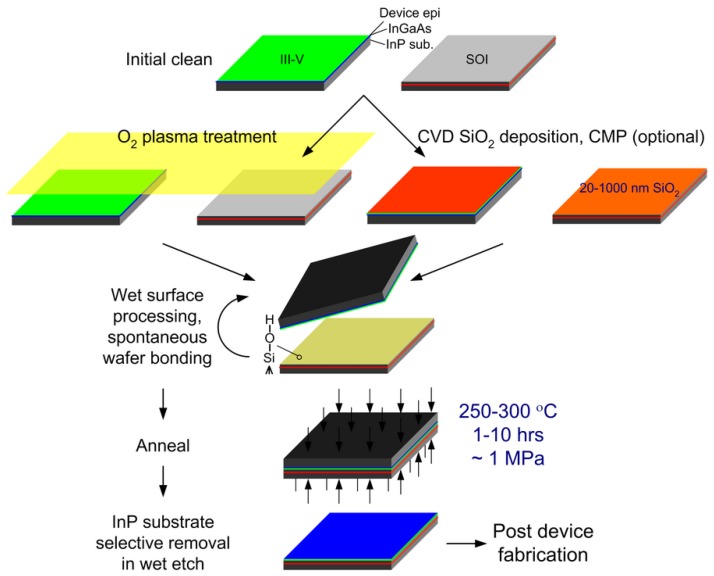
Schematic process flow for O_2_ plasma-assisted and SiO_2_ covalent wafer bonding.

Figure 1 shows the schematic process flow of the O_2_ plasma-assisted and SiO_2_ covalent wafer bonding. After rigorous sample cleaning and close microscopic inspection, the native oxide on SOI and InP are removed in standard buffered HF solution and NH_4_OH (39%), respectively, resulting in clean, hydrophobic surfaces. In O_2_ plasma-assisted process, the samples then undergo an O_2_ plasma surface treatment to grow an ultra-thin layer of plasma oxide (~15 nm) [14], which leads to very smooth (RMS roughness <0.5 nm) hydrophilic surfaces [15]. The Si-O-Si bonds of the oxide (SOI side) are found to be more reactive than conventional oxides formed in a standard RCA-1 cleaning process or other hydrophilic wet-chemical treatment, and have a higher readiness to break and form new bonds [16]. O_2_ energetic ion bombardment also acts as a final cleaning step, efficiently removing hydrocarbons and water related species on the sample surface. 

For SiO_2_ covalent direct bonding, a clean hydrophilic surface comes by depositing SiO_2_ on both surfaces, (e.g., plasma-enhanced chemical vapor deposition (PECVD) SiO_2_) on non-Si materials or thermally grown SiO_2_ on Si. If surface RMS roughness exceeds 1 nm, which is believed to be the maximum surface roughness for strong direct wafer bonding in the literature [16], chemical mechanical polishing (CMP) is routinely employed to improve surface topography [17]. Both bonding methods require a final activation step to passivate the two surfaces with a high density of polar hydroxyl groups (-OH), bridging bonds between the mating surfaces, enabling spontaneous bonding at room temperature. O_2_ plasma-treated samples are simply dipped in deionized water and blow-dried or placed in a vaporized NH_4_OH environment. Thick SiO_2_-covered samples are boiled in diluted RCA-1 solution at 75 °C for 10 minute, a step to clean and form an Si-OH-passivated surface, then blow dried. Similar O_2_ plasma treatment [18] and a quick dip in very dilute HF solution (0.025%) for 1 minute [12,18] has also been found to be very helpful at suppressing interfacial voids and enhances the ultimate bonding strength. Following immediate physical mating typically in air at room temperature, the bonded sample is annealed at 300 °C with external coaxial pressure (1–2 MPa) for an hour or more to form strong covalent bonds through the polymerization reactions in Equations (1) and (2). M in Equation (1) refers to metals with relative high electronegativity (e.g., In, P) [19]. After annealing and cooling, the InP substrate is selectively removed in a 3HCl:1H_2_O solution at room temperature to leave thin (<2 μm) InP-based epitaxial layers on Si.

Si-OH + M-OH ➔ Si-O-M + HOH (g)
(1)

Si + 2H_2_O ➔ SiO_2_ + 2H_2_ (g) (2)

### 2.2. Solutions to outgassing in hydrophilic bonding

Equations (1) and (2) represent the fundamental polymerization reactions in Si-based hydrophilic direct bonding [1], *i.e.*, inorganic-to-inorganic bonding methods in this paper. The generated gas byproducts of H_2_ and H_2_O [20], have been experimentally proven to be the major trapped gases at the bonding interface [21,22]. A significant amount of gas formation and desorption of 2–3 monolayers of water molecules at the bonding interface of hydrophilic wafers after room temperature mating, plus gaseous hydrocarbon from organic surface contamination during the anneal, can lead to high internal pressure [22], subsequently resulting in local debonding, *i.e.*, interface void formation. Typically, gas molecules, especially at high temperatures with a small atom size such as H_2_, can diffuse out through the micoroughness at the interface gradually or enter quickly through a porous medium (such as SiO_2_). Interfacial voids can also be filled up due to native or thermal oxide viscous flow at high temperatures (>800 °C) [23]. An elevated temperature anneal is therefore naturally preferred for its void-free, strong bonding and its processing simplicity with no need of prebond surface activation. For example, manufacturing of commercial wafer-bonded silicon-on-insulator (SOI) wafers up to 300 mm in diameter [9]. However, prohibition of elevated temperature anneals results in outgassing as a major issue in all low-temperature hydrophilic bonding [24]. 

Embedding a thick layer of porous material such as thermal SiO_2_ or PECVD dielectrics has been reported as an efficient outgassing medium for H_2_O and H_2_ diffusion and absorption [11,24], which is a motivation of using SiO_2_ covalent bonding here. However, it is not applicable for situations where integration with a high proximity of two mating materials is needed, or optical, electrical or thermal interactions between mating materials are desired. Outgassing effectiveness is also limited when an interfacial layer becomes thin (e.g., <500 nm). 

**Figure 2:**
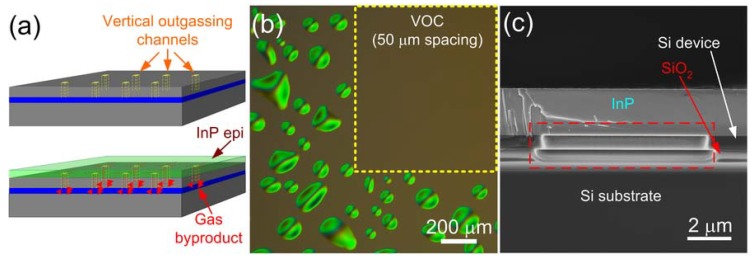
(a) Schematic cartoons of vertical outgassing channels (VOCs) on the SOI substrate before (top) and after (bottom) contacting InP epitaxial layers. (b) Normaski microscopic images of an InP-to-SOI bonded pair after 300 °C anneal for 30 min, showing noticeable contrast between VOC region (yellow dash line box) and non-VOC region. (c) SEM cross-sectional view of VOCs with InP epitaxial layers bonded on top, showing intimate contact with no deformation or delamination. Red dash line box highlights a single VOC.

A vertical outgassing channel (VOC) design is therefore developed to tackle this outgassing issue for O_2_ plasma-assisted bonding, where the interfacial oxide layer is typically less than 15 nm thick. As illustrated in the cartoon in Figure 2(a), VOCs are essentially an array of holes with a few micrometers in diameter and etched through the top Si device layer to the buried oxide (BOX) layer prior to contact with the III-V material. The generated gas byproduct molecules, plus a small amount of trapped air molecules including any gaseous impurities, can migrate to the closest VOC, and can promptly be trapped inside by VOCs and start being absorbed by BOX. They may also gradually diffuse out through the BOX layer, due to its open network of only 43% of the occupied lattice space [25] and its large diffusion cross-section, generally 0.3–3 μm thick. 

Figure 2(b) shows the Normaski optical microscopic image of 2 μm-thick III-V epitaxial layers transferred onto SOI substrate. VOCs with an optimized channel spacing 50 μm and size 8 × 8 μm^2^ [26] are only patterned in certain area of SOI sample highlighted by yellow dash line box. One can notice clear contrast between void-free VOC region and non-VOC region, where a great number of interfacial voids are evenly distributed. SEM cross-sectional view of a corresponding VOC with thin III-V expitaxial layers bonded on top is illustrated in the red dash line box of Figure 2(c), demonstrating intimate contact of III-V and Si with no III-V deformation above VOC. The absence and undercut of BOX is due to the wet etch of SiO_2_ hard mask in HF solution after transferring the VOC pattern from the hard mask to the Si device layer, and has shown no negative impact on the effectiveness of the VOC outgassing. 

Bonding strength (or bonding surface energy) is measured by the conventional crack-opening method [27]. An attempt was made to wedge a thin blade into the bonding interface to separate the III-V from Si, so that one could calculate the bonding strength from the distance of separation. However, the consistent cracking of the InP substrate from both bonding methods indicates the likelihood of the bonding surface energy higher than the fracture energy of bulk InP (0.7 J/m^2^), a similar case that Maszara reported in measuring hydrophilic Si-Si bonds [28]. Over 2.5 J/m^2^ surface energy was measured accurately in a SiO_2_ covalent bonded sample where the III-V was replaced by another Si wafer while keeping everything else constant [18]. 

### 2.3. Wafer-scale integration

**Figure 3:**
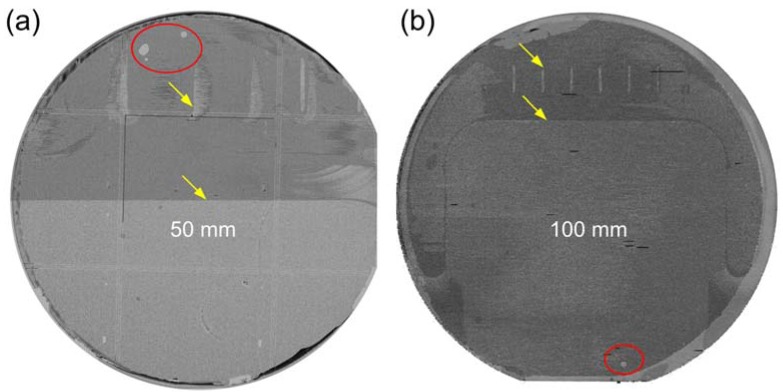
SAM images of (a) 50 mm and (b) 100 mm InP-to-Si bonded wafers. Interfacial voids are highlighted in red circles and yellow arrows indicate the horizontal (vertical in the image) scan lines and a pattern from the wafer chuck related to the tool.

Compared to heteroepitaxial growth, wafer bonding embraces a major advantage of low-cost *wafer-scale* integration, as long as bonding criteria are satisfied. As mentioned before, up to 300 mm high-quality SOI wafers for microelectronics are routinely manufactured by wafer bonding [9]. Upon solving outgassing issues in low-temperature bonding, wafer-level III-V-to-Si bonding is also attainable. For typical small bonded pieces (~cm^2^), Normaski optical microscopy is sufficient to spot any interfacial voids as small as sub-μm in diameter after removing the thick InP substrate. High-resolution automatic scanning acoustic microscopy (SAM) is more convenient for wafer-scale interface inspection. Figure 3(a) and Figure 3(b) show wafer-scale SAM images of 50 and 100 mm in diameter O_2_ plasma-assisted bonded wafers, respectively, with sub-μm microscopic resolution (X-axis: 0.5 μm, Y-axis: 0.25 μm, Z-axis: 0.5 μm) [14]. Only few relatively large voids (highlighted by red circles) are observed. They are close to the wafer edge and likely from surface particles collected during manual wafer handling. No uniformly distributed gas byproducts-resulted voids are found, which is compatible with inspection in Normaski microscopy in selected areas [15]. Void-free bonding is therefore achieved in >99% of the area. The misleading contrast and some vertical lines highlighted in the SAM images are from the wafer chuck related to the SAM tool. 

**Figure 4:**
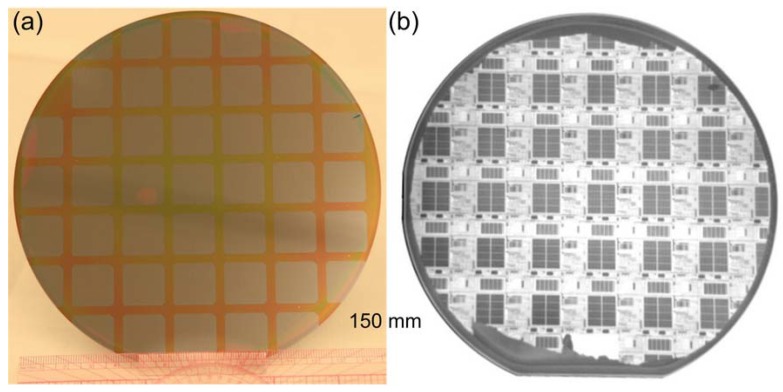
(a) Photograph of processed 150 mm diameter O_2_ plasma-assisted bonded wafer showing individual device dies. (b) IR image of 150 mm SiO_2_ covalent bonded wafer with CMOS devices successfully transferred.

Up to 150 mm in diameter III-V-to-Si bonding, presently the largest available III-V epitaxial wafer, are demonstrated as well using O_2_ plasma-assisted and SiO_2_ covalent bonding methods. Figure 4(a) shows the photo of 150 mm in diameter thin III-V epilayers transferred onto the SOI by O_2_ plasma-assisted bonding process. The processing time does not increase with increasing wafer size, provided that cleaning time does not vary much, which indicates the advantage of wafer-scale integration through wafer bonding. Over 98% area transfer and mirror-like III-V surface with a typical root mean square (RMS) roughness of 0.6–0.7 nm was demonstrated [15,26]. Further III-V back-end process proceeded to fabricate race-track ring resonator lasers on SOI substrate. After putting a layer of 300 nm plasma-enhanced chemical vapor deposition (PECVD) SiN_x_ at 260 °C, the wafer underwent standard projection photolithography and dry etch patterning of the racetrack ring laser layout arrayed on each device die (2 × 2 cm^2^). Wet etching then removed a surface InGaAs layer in exposed areas, giving us access to PL response from active region [14]. No extra delamination was observed during thermal cycling and wet processing, indicating robust III-V-to-Si bonding. A 150 mm diameter SiO_2_ covalent bonded wafer is also exhibited in Figure 4(b), where thin Si devices were transferred onto InP substrate for CMOS-on-III-V mixed material integration [17]. The infrared (IR) image in Figure 4(b) shows void-free interface and transferred SOI CMOS circuit.

### 2.4. Epitaxial transfer quality characterization in wafer-scale integration

**Figure 5:**
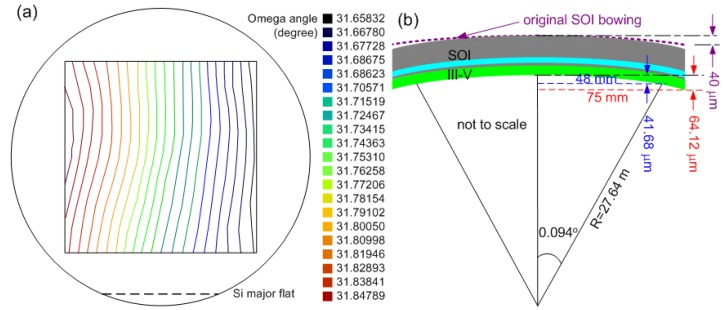
(a) Wafer warpage contour composed of 81 Omega scans at a 9 × 9 matrix with 12 mm step size. (b) Schematic warpage of bonded wafer pair with a bowing of 64.12 μm with a corresponding surface radius of 27.64 m based on the largest omega angle difference measured in (a).

In order to study the inevitable thermal stress from 300 °C anneal due to mismatch of InP and Si thermal expansion coefficients, epitaxial transfer quality of the 150 mm diameter bond is characterized by high-resolution x-ray diffraction (XRD) rocking curve measurement. The (004) diffraction peak from 1.5 μm-thick InP cladding layer is used as the reference. A wafer map composed of 81 Omega scans (0.2°) to a 9 × 9 matrix (step size: 12 mm) is illustrated in Figure 5(a) for determining the surface warpage, so that bonding-induced strain can be extracted later. A maximum peak position shift of 0.188° in Omega angle at two points along the horizontal direction (96 mm long) results in a bowing of 41.68 μm, leading to an approximate 64.12 μm warpage across the 150 mm diameter wafer as shown in Figure 4(b). This is comparable to the maximum 80 μm bow of the commercial Unibond^TM^ SOI substrate from SOITEC. Thermal stress can be calculated through Equation (3),

(3) $$\sigma_{f}=\frac{E_{s}{t_{s}}^{2}}{6\rho t_{f}(1-\nu_{s})}$$

where *E_s_*, *v_s_* and *ρ* represent the Young’s modulus (*E_Si_* = 98 GPa), Poisson ratio (*v_Si_* = 0.29) and surface curvature (*ρ* = 64.12 μm) of Si substrate since all stress is concentrated in III-V after InP substrate removal. With a 2 μm thick III-V epitaxial layer (*t_f_* = 2 μm) and a 625 μm thick SOI wafer (*t_s_* = 625 μm), and taking the measured original SOI wafer bowing of 40 μm into account, the calculated stress is 17 MPa tensile, much smaller than typical 200 nm PECVD SiO_2_ or SiN_x_-induced thin film stress in a range of 20–200 MPa. It is noted that the observed warpage is parallel to the Si major flat direction, because the removal of the Si wafer at the wafer major flat resulted in a weak direction that is easiest to bend. The same wafer map measurement with a wafer rotated 90° at the measurement plane (data not shown) confirms the warpage direction. XRD characterization therefore indicates a mirror-like, flat, low-strain epitaxial transfer. 

## 3. Adhesive Wafer Bonding Technology 

Next to the direct bonding approach described in the previous section, adhesive bonding can also be used to transfer III-V epitaxial layers onto a SOI waveguide circuit. We will start this section with a literature review of the various types of thermosetting adhesives, to come to the selection of DVS-BCB for this particular application. Thermosetting adhesives are required, since the post-bonding processing temperatures for III-V optoelectronic components can go up to 400 °C, excluding thermoplastic and elastomer bonding agents. The literature review is not exhaustive. An extensive review of adhesive wafer bonding can be found in [29]. We will discuss the use of polyimides, epoxies, spin-on-glasses, photoresist, SU-8 and DVS-BCB. In [30], Dupont polyimide PI2610 was tried as an adhesive, to bond two 100 mm diameter silicon wafers. Large unbounded areas resulted due to the presence of voids at the bonding interface. This is attributed to the creation of byproducts during the imidization-process, which get trapped at the bonding interface, and to the large volume shrinkage upon cure, which might affect the adhesion due to high stresses in the polymer coating. In [31], a silver-loaded conductive epoxy (EpoTekH20E) was used to bond 50 mm in diameter GaAs wafers. The bonding was performed at 120 °C. In this experiment, post-bonding processing was performed: the original GaAs substrate was removed using a combination of mechanical grinding and chemical etching and LEDs were processed in the bonded epitaxial layer. The thickness of the bonding layers used in the experiments was not specified. Spin-on glass (SOG) was used in [32] to bond 1 cm^2^ InP/InGaAsP vertical-cavity surface-emitting laser (VCSEL) epitaxial layer structures to a silicon substrate. The bonding layer thickness varied from a few tens of nanometer to 300 nm by varying the pressure exerted on the wafer stack. In this case, the spin-on glass was spin coated very slowly in order to remain liquid after spin coating. After mating of the surfaces, a bonding pressure between 10 and 60 kPa was applied on the wafer stack. The wafer stack was cured at 400 °C. Two types of spin-on glass were used for the experiments: all samples bonded with silicate SOG tended to separate when trying to cut them using a dicing saw. Experiments with siloxane SOG, spin-on glass with a higher organic content, were successful. This difference is attributed to the large shrinkage of the silicate SOG upon cure, resulting in a large film stress, and the low cracking resistance of the silicate SOG. In [33], 100 mm and 150 mm silicon wafers were bonded using SOG. A high bonding strength was measured after room temperature bonding, due to the chemistry at the SOG/silicon interface, resulting in bonding at room temperature. Thermal annealing was performed at 200 °C for 18 hours. Defect-free bonded wafer pairs were obtained. This process was also used for the fabrication of GaAs/silicon heterostructures. Annealing temperatures were limited to 225 °C in order to avoid debonding and shattering of the GaAs wafer. After thinning the GaAs substrate to a remaining thickness of 10 µm, the bonded pair was heated to 450 °C without debonding or void generation at the bonding interface. In [30], 1.5 to 2.3 µm thick (thermosetting) Shipley photoresist S1818 layers were used to bond 100 mm silicon wafers. The bonding was performed at 120 °C using 300 kPa bonding pressure. In the experiment, no post-bonding temperature excursions were made. The reported bonding strength was low compared to other thermosetting adhesives used, like polyimide and DVS-BCB. In [34], 20–45 µm thick SU-8 epoxy layers (thermosetting) were used to bond a 100 mm silicon wafer onto a 100 mm Pyrex wafer. The bonding was performed at 120 °C using 300 kPa bonding pressure. In the experiment, no post-bonding temperature excursions were made. From this review, it is clear that a whole myriad of thermosetting adhesives can be used for wafer bonding applications.

In the literature, there are many reports available on the use of DVS-BCB (divinylsiloxane-bis-benzocyclobutene, a thermosetting polymer) as an adhesive bonding agent [29,30,35,36,37,38]. DVS-BCB is superior in terms of high bonding strength and bonding quality (due to the fact that no by-products are created during curing), its high degree of planarization and its resistance to all sorts of chemicals used in III-V processing. In the subsequent section, we will outline the DVS-BCB adhesive wafer bonding process for III-V epitaxial layer transfer to a silicon-on-insulator waveguide circuit. Some important properties of DVS-BCB are outlined in Table 1 [35].

**Table 1:** Overview of the DVS-BCB die-to-wafer bonding process [35].

*Electrical properties*	
Dielectric constant	2.5 at 10 GHz
Dissipation factor	0.002 at 10 GHz
Breakdown voltage	5.3 MV/cm
*Optical properties*	
Refractive index	1.543 at 1.55 µm
Optical loss	<0.1 dB at 1.55 µm
*Mechanical properties*	
Tensile modulus	2.9 GPa
Intrinsic stress	28 MPa
Tensile strength	89 MPa
Poisson ratio	0.34
Shrinkage upon cure	0.05
*Thermal properties*	
Glass transition temperature	>350 °C
Thermal conductivity	0.29 W/mK
Thermal expansion coefficient	42 ppm/K
*Other properties*	
Planarization	Very good
Moisture uptake	Very low

### 3.1. DVS-BCB adhesive die-to-wafer bonding

**Figure 6:**
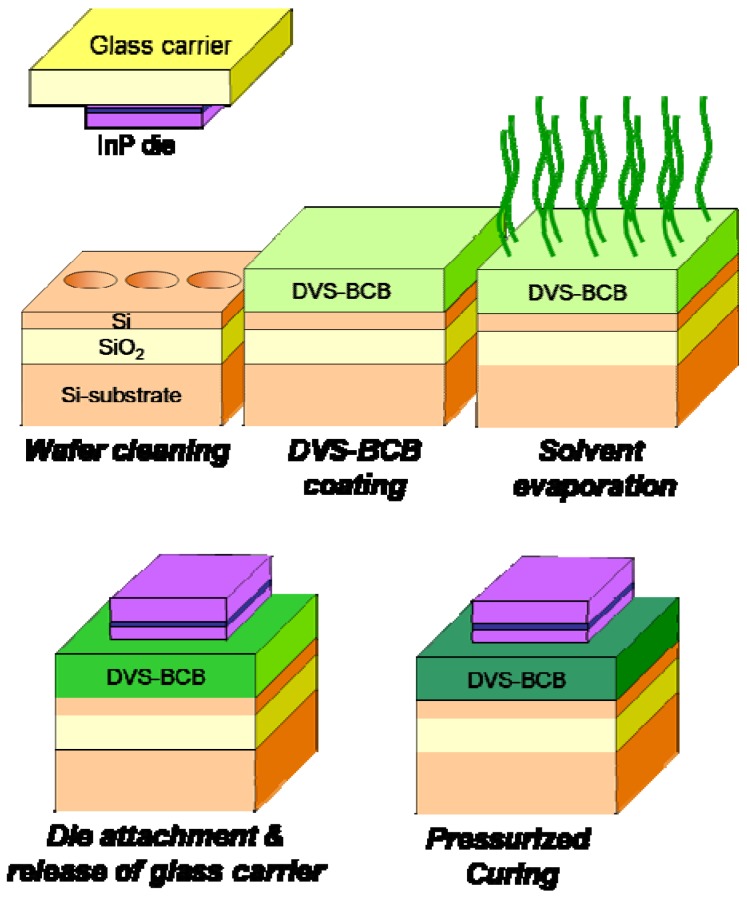
Overview of the DVS-BCB die-to-wafer bonding process.

The DVS-BCB adhesive die-to-wafer bonding process is schematically outlined in Figure 6. The InP/InGaAsP epitaxial layer structures are cleaved into individual dies (typical dimensions are in the range of 25 mm^2^−100 mm^2^) and are temporarily mounted on a glass carrier using either a thermoplastic adhesive or thermal release tape. The mounting on a glass carrier serves two purposes: first of all it allows easy handling of the III-V dies during cleaning of the die surface. Secondly, the glass carrier allows mounting multiple III-V dies at the same time, thereby allowing multiple die-to-wafer bonding. The most important part of the die-to-wafer bonding procedure consists of the cleaning of the silicon-on-insulator waveguide wafer and III-V dies. 

**Figure 7:**
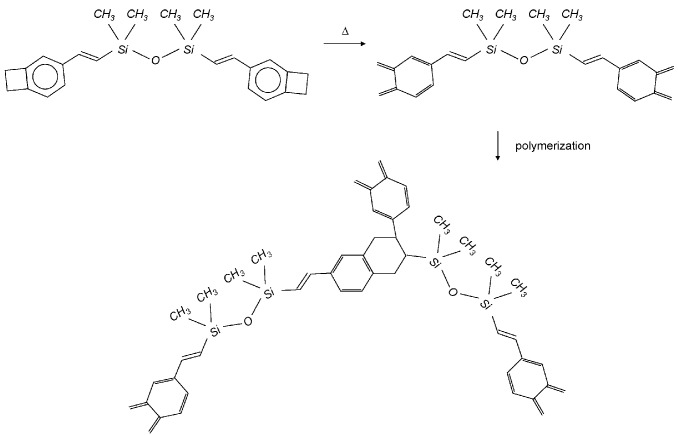
Polymerization reaction of the DVS-BCB monomer.

The SOI waveguide wafer is cleaned using a Standard Clean 1 solution (*i.e.,* a mixture of NH_4_OH, H_2_O_2_ and H_2_O). This cleaning step lifts off particles from the SOI wafer surface and renders the surface hydrophilic. The III-V die is cleaned, while mounted on the glass carrier wafer, by selectively removing a sacrificial InP/InGaAs layer pair using HCl and H_2_SO_4_:3H_2_O_2_:H_2_O, respectively. This step removes organic contamination from the III-V die surface (which is present due to the mounting of the die on the carrier) and also lifts off particles from the die surface, which are generated during the cleaving of the III-V die. Following cleaning, an adhesion promoter is spin coated on the SOI waveguide circuit (AP-3000, Dow Chemicals), after which the DVS-BCB oligomer solution is spin-coated on the III-V die. Since ultra-thin bonding layers (typically sub 100 nm) are required for a good optical and thermal coupling between the III-V and SOI in the hybrid device platforms in Section 4, commercially provided DVS-BCB oligomer solutions (Dow Chemicals) need to be diluted using mesitylene, to achieve the required bonding layer thickness. Although the topography of the SOI waveguide wafer (220 nm) is larger than the required spacing between the top of the SOI waveguide and the III-V epitaxial layer structure, still a good planarization can be achieved. After spin coating, the SOI is heated to 150 °C for 1 minute in order to evaporate the remaining mesitylene solvent in the spin coated DVS-BCB film. This is required to avoid the generation of voids at the bonding interface. After the evaporation of the mesitylene, the III-V die is attached, epitaxial layers down, to the SOI waveguide circuit. This can be done either in cleanroom air (manually) or in the vacuum chamber of a commercial automatic wafer bonding tool. The latter option gives better results in terms of uniformity and repeatability. After die attachment at 150 °C, the III-V/DVS-BCB/SOI stack is cured at 250 °C. Upon curing, the benzocyclobutene ring thermally opens to form o-quinodimethane. This very reactive intermediate readily undergoes a so-called Diels-Alder reaction with an available vinylsiloxane group to form a three-dimensional network structure as is shown in Figure 7 [35]. As is clear from this reaction mechanism, no byproducts are created during the polymerization. Since no by-products are created, the result is a void free bond. 

**Figure 8:**
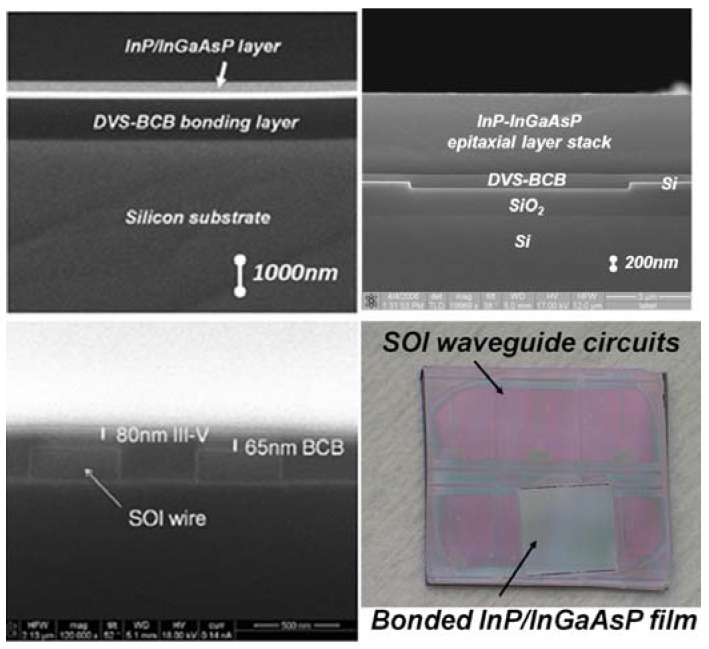
SEM cross-section images of transferred III-V layer stacks and a top-down view.

After bonding, the original InP growth substrate is removed using grinding and selective wet chemical etching using the same 3HCl:H_2_O solution and an InGaAs etch stop layer. This leaves the epitaxially grown III-V layer stack attached to the SOI waveguide circuit. SEM cross-sections of the fabricated layer stacks and a top-down view of a transferred epitaxial layer to a silicon-on-insulator substrate are shown in Figure 8. This figure illustrates the versatility of the DVS-BCB adhesive bonding process, since a whole range of bonding layer thicknesses (from 50 nm up to a few micrometer) can be achieved.

The amount of strain that is generated in the inorganic-organic layer stack is also studied. This strain originates from the difference in thermal expansion coefficient between the silicon substrate, the III-V epitaxial layer and polymer interfacial layer. The strain of the bonded layer stack as a function of temperature (after curing the DVS-BCB bonding layer at 250 °C for 1 hour) was mapped out by monitoring the bow of the wafer stack. 

**Figure 9:**
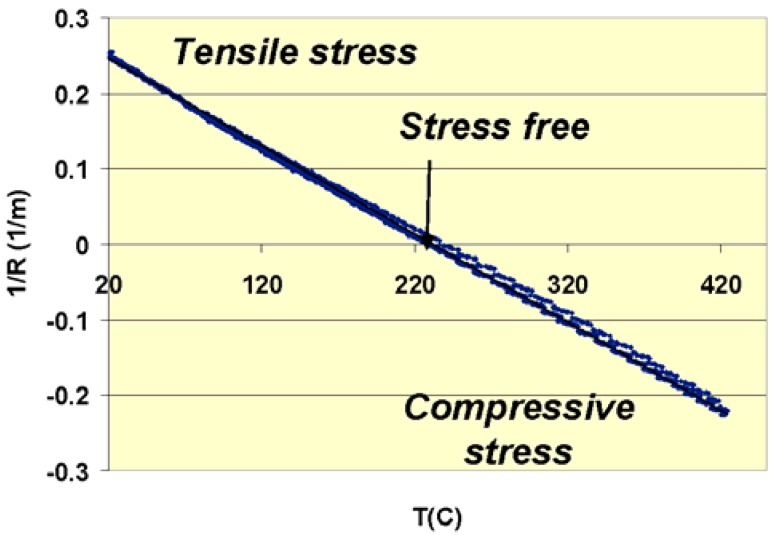
Curvature of the bonded stack as a function of temperature.

Similar to the wafer bowing in Figure 5, the curvature of the adhesive bonded stack reveals the strain in the transferred epitaxial layer. A linear dependence as a function of temperature is observed in Figure 9. At room temperature, the III-V epitaxial film is under tensile stress. Around 230 °C, the stack is stress-free, since around this temperature during the curing cycle, the DVS-BCB fixes the III-V layer. Above this temperature compressive strain is observed in the transferred epitaxial layer. From this curvature, the remaining strain in the transferred epitaxial layer can be calculated. Stress levels at room temperature up to 40 MPa are obtained, which correspond to a strain of 0.04%. This is sufficiently low from an optical point of view as the influence of the stress on the band shifting is acceptable. Comparing the simulation results with the stress levels needed to induce dislocations in the layer structure, elevating the temperature after bonding to temperatures higher than 400 °C could create dislocations in the InP layer, which should be avoided.

## 4. III-V-on-Si Photonic Device Platforms

Two recently demonstrated III-V-on-Si device platforms, primarily for building photonic integrated circuits on silicon substrate, have been developed. One is named as hybrid silicon platform, which is based on the O_2_ plasma-assisted direct bonding technique and developed by a joint effort between University of California-Santa Barbara (UCSB) in the U.S.A. and Intel Corporation. The other is called heterogeneous III-V/SOI platform, which is based on the adhesive wafer bonding technique and developed by Ghent University in Belgium. Unlike the conventional way to attach the bulky (>100 μm thick) individual III-V lasers onto finished Si circuits, they both involve transfer of as-grown thin (<2 μm thick) crystalline III-V thin film to a SOI host substrate. The Si is typically patterned prior to the transfer, and the III-V films are processed after transfer allowing for the use of standard lithography based patterning techniques to fabricate III-V optoelectronic devices. A fraction of light generated in III-V is then evanescently coupled to the Si waveguide underneath.

### 4.1. Hybrid silicon platform 

**Figure 10:**
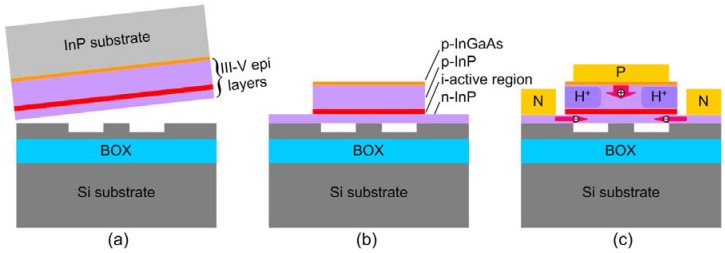
A simplified fabrication process to form the hybrid Si device platform. (a) Bonding of the III-V wafer to the patterned SOI wafer. (b) InP substrate removal and mesa etching. (c) Current confinement and metal contact formation.

Figure 10 highlights the critical steps to form a hybrid Si platform. The hybrid structure is comprised of III-V epitaxial layers transferred to a SOI waveguide through a low-temperature, O_2_ plasma-assisted wafer bonding process (Figure 10(a)). Upon removing the thick InP substrate selectively, the mesa structure to enable a carrier injection scheme similar to VCSEL devices is then formed on the III-V region by standard photolithography and etching (Figure 10(b)). Fabry-Perot (FP), Distributed-Feedback (DFB), Distributed Bragg reflector (DBR) and ring resonator structures can be realized easily to provide feedback for lasing. Typically, the III-V mesa width is larger than the Si waveguide (1–2 μm) so that the transverse mode confinement is determined by the SOI waveguide and not the III-V mesa. This eliminates any issues with alignment of the III-V etch to the SOI etch. Amplifiers and lasers have a wide III-V mesa (12 μm−14 μm) for better heat conduction and mechanical strength while a narrow III-V mesa (2 μm−4 μm) is chosen for detectors and modulators for high speed operation with a reduced capacitance. H^+^ proton implantation is used if carrier confinement or electrical isolation between integrated devices is necessary. In the case of a wide III-V mesa, proton implantation is employed to confine the current flow for better overlap with the optical mode (Figure 10(c)). Detailed fabrication steps can be found in Ref. [39]. The general structure of III-V layers consists of a p-type InGaAs contact layer, a p-type InP cladding, an optional p-type separated confinement heterostructure (SCH) layer, an undoped multiple quantum well layer, n-type contact layer, and n-type superlattice bonding layers. The employment of superlattice bonding layers is for reducing and blocking the TEC mismatch-induced defects propagating into the active region [14,40]. Examples of epitaxial structures for electrically-pumped lasers can be found in Refs. [39,41]. 

**Figure 11:**
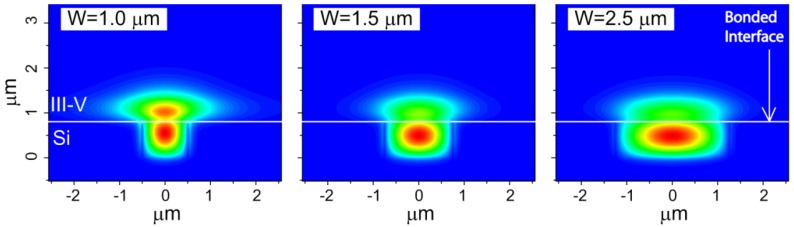
Mode profiles with different waveguide widths. The height of the Si waveguide is fixed at 0.7 μm [42].

Due to the similar refractive index of Si and III-V materials, the optical mode in this hybrid waveguide lies both in the Si waveguide and III-V mesa. A unique feature of this platform is the flexibility to widely adjust optical confinement factors in Si and III-V layers. A high confinement factor is useful for low threshold in lasers, high gain, low noise amplification in optical amplifiers and high quantum efficiency in photodetectors. A low confinement factor is useful for high power lasers, high saturation current amplifiers and high power photodetectors. In many cases, the best performance can be obtained by changing the confinement factor along the length of the device. For example, changing the confinement factor from high to low results in high gain, low noise, high saturation power optical amplifiers. Changing the confinement factor from low to high is useful for high saturation current, high quantum efficiency photodiodes. This can be implemented in the hybrid silicon platform by changing the width of the waveguide (Figure 11; calculated from Beam Propagation Method (BPM) simulations). The Si confinement factor is an important parameter in determining coupling efficiency when the device is integrated with Si passive devices. Mode profiles from different waveguide widths (W = 1, 1.5 and 2.5 μm) in Figure 11 show that narrowing the Si waveguide pushes the mode up to the III-V, resulting in a large III-V confinement factor, while a wide Si waveguide accommodates a larger portion of the optical mode. A taller Si waveguide also leads to larger Si confinement factor. This unique characteristic therefore allows different confinement factors in different regions of the hybrid waveguide for the same III-V epitaxial structure, catering to the requirements of different components on the same chip. 

### 4.2. Heterogeneous III-V/SOI platform 

**Figure 12:**
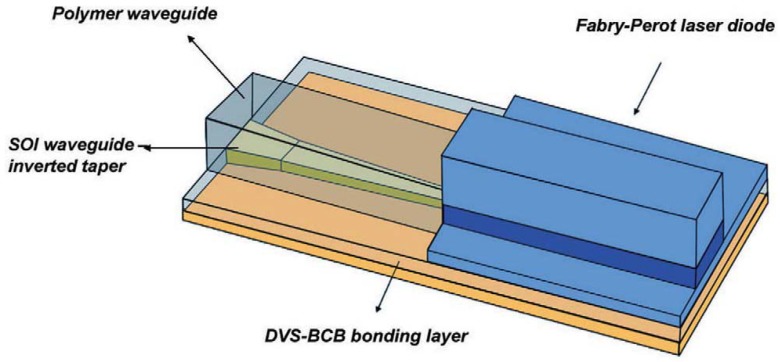
Schematic of the layout of the optical coupling scheme for efficient and fabrication tolerant coupling between a bonded Fabry-Perot laser diode and an underlying SOI waveguide circuit using an inverted adiabatic taper approach [44].

Another hybrid III-V-on-Si platform recently developed by Ghent University possesses similar device structure, though the III-V material and the Si waveguide perform relatively independent functions [43]. A three-dimensional schematic is shown in Figure 12 to depict the photon generation, optical feedback and coupling to the Si waveguide below [43]. The III-V epitaxial layer transfer is achieved by thermosetting polymer divinylsiloxane-benzocyclobutene (DVS-BCB) adhesive bonding [43] as discussed in section 3.1. A typical DVS-BCB layer is on the order of several hundreds of nanometers with a refractive index ~1.5 at λ = 1.55 μm. A relatively thick low-index medium between III-V and Si prevents photons generated in the III-V active region from coupling into the Si waveguide instantly. Lasing is achieved through the gain provided by a III-V active region and reflection at the etched laser facets. As the stimulated emission leaves the edge of the laser diode, an additional coupling structure is required for efficient coupling to the SOI waveguide. An optimal adiabatic inverted taper structure is employed to achieve good coupling efficiency and fabrication tolerance. The concept of the inverted adiabatic structure is to butt-couple the bonded laser diode to a polymer waveguide, after which the optical mode is gradually transformed into that of the SOI waveguide by increasing the cross-sectional area of the Si waveguide. The polymer waveguide is self-aligned to the laser ridge, eliminating a possible source of coupling efficiency reduction arising from the misalignment between the waveguides. The Si inverted taper structure is buried underneath the polymer waveguide. The inverted taper tip width has to be sufficiently small in order for the fundamental optical waveguide mode at the tip to resemble the waveguide mode of the polymer waveguide closely [44]. It is noted that recent work has enabled the 50 nm thick DVS-BCB layer for strong coupling. That results in more similar operation principle to hybrid silicon platform above. The formation of III-V mesa and electrodes are similar to the platform. 

The two III-V-on-Si hybrid platforms which are enabled by inorganic-inorganic or inorganic-organic wafer bonding techniques have led to a series of active components for Si photonic integration circuits recently. They include Fabry-Perot cavity [39,44], racetrack ring [41], mode-lock [45], micro-disk [46], distributed feedback [47], distributed bragg reflector [48] and micro-ring [49] lasers, amplifiers [50], PIN [51] and metal-semiconductor-metal [52] photodetectors, electroabsorption [53], Mach-Zehnder interferometer phase [54] and micro-disk [55] modulators, high-speed switches [56], *etc.* More advanced integration circuits have also started being demonstrated [52,57]. It is noted that in addition to the UCSB-Intel research team and the European research team led by Ghent University, several groups worldwide have also made important contributions to this hybrid integration approach to active Si photonics [58,59,60,61]. This is important to developing multi-functional Si photonic integrated chips for a variety of immediate and emerging applications.

## 4. Conclusions

In this paper we have reviewed low-temperature inorganic-to-inorganic (*i.e.*, O_2_ plasma-assisted and SiO_2_ covalent direct bonding) and inorganic-to-organic (*i.e.*, DVS-BCB adhesive bonding) wafer bonding methods. Both techniques successfully enabled two similar III-V-on-Si hybrid platforms for high-performance Si photonic integrated circuits. Table 1 summarizes characteristics associated with the two bonding methods. Both methods can generate strong, low stress and stable bonding under low temperature process requirement. Compared to direct bonding, adhesive bonding doesn’t require special surface treatment and is able to tolerate some surface topography, depending on interfacial polymer chemistry and layer thickness. This results in a relatively simple process. Direct bonding, however, embraces the inherent advantage of better integration proximity, which benefits optical coupling and heat transportation between dissimilar materials for the hybrid III-V-on-Si photonic integration platform in particular, provided that the outgassing problem is sufficiently minimized or eliminated. Both techniques have paved a way to integrate dissimilar materials without compromising their own properties, and are by no means restricted to III-V-on-Si integration here. The bonding technique adoption is determined by application and specific device design eventually.

**Table 1:** Basic bonding characteristic of direct bonding and adhesive bonding.

Bonding characteristic	Molecule bonding	Adhesive bonding
Bonding strength (<400 °C)	High	High
Process complexity	Medium	Low
Tolerance to surface defects, roughness and contamination	Low	High−medium
Bonding-induced strain	Low	Low
Integration proximity	High	High–medium
Intrinsic outgassing problem	High	Low
Uniformity	High	High–medium
Stability	High	High
Scalability	High	High
